# Introduction of Depot Buprenorphine in Slovenian Prison Service

**DOI:** 10.1192/j.eurpsy.2026.10886

**Published:** 2026-07-31

**Authors:** M. Delic

**Affiliations:** Center for Treatment of Drug Addiction, University Psychiatric Clinic Ljubljana, Ljubljana, Slovenia

## Abstract

**Introduction:**

Drug use disorders (DUD) remain among the most prevalent and challenging public health concerns in European prisons. Opioid agonist treatment (OAT) is effective in managing opioid dependence. In Slovenia, all patients with opioid use disorder have access to methadone, oral buprenorphine, and depot buprenorphine while in custody. Most patients are treated with oral buprenorphine; however, diversion of buprenorphine tablets has been reported in all Slovenian prisons. Long-acting injectable depot buprenorphine has been shown to be safe and effective, while also supporting longer periods of abstinence and providing a strong foundation for behavioral change. Administered subcutaneously on a weekly or monthly basis, depot buprenorphine represents a promising approach to address the problem of diversion.

**Objectives:**

To present the challenges of managing opioid dependence in prisons and to evaluate the role of depot buprenorphine in improving treatment outcomes.

**Methods:**

We analyzed data on OAT in one of Slovenia’s largest prisons and described the process of introducing depot buprenorphine.

**Results:**

Ljubljana Prison is the second-largest correctional facility in Slovenia. In the first eight months of 2025, 71 men aged 18 years or older with a diagnosis of opioid use disorder (ICD-11) were serving custodial sentences. Of these, 24 were treated with methadone and 46 with oral buprenorphine. All were offered the option of switching to depot buprenorphine; 11 accepted. Four patients discontinued treatment due to injection-site swelling, and one due to a severe rash.

**Conclusions:**

The introduction of depot buprenorphine in prison settings may improve patient safety, strengthen therapeutic relationships, and reduce the diversion of opioid medications. Uptake, however, remains limited due to concerns about side effects and reluctance to switch from established oral regimens. Motivational techniques are therefore essential to support patients in making this transition, enhancing adherence, and maximizing treatment outcomes. Importantly, depot buprenorphine also ensures continuity of care and provides an additional layer of safety after release, when the risk of relapse and overdose is highest.

**Disclosure of Interest:**

None Declared

